# The association between physical activity and atrial fibrillation applying the Heaviside function in survival analysis: the Multi-Ethnic Study of Atherosclerosis

**DOI:** 10.4178/epih.e2017024

**Published:** 2017-06-18

**Authors:** Yaser Mokhayeri, Seyed Saeed Hashemi-Nazari, Mohammad Ali Mansournia, Hamid Soori, Soheila Khodakarim

**Affiliations:** 1 Department of Epidemiology, School of Public Health, Shahid Beheshti University of Medical Sciences, Tehran, Iran; 2 Safety Promotion and Injury Prevention Research Center, Shahid Beheshti University of Medical Sciences, Tehran, Iran; 3 Department of Epidemiology and Biostatistics, School of Public Health, Tehran University of Medical Sciences, Tehran, Iran

**Keywords:** Exercise, Atrial fibrillation, Arrhythmias, Walking speed

## Abstract

**OBJECTIVES:**

Although the effect of physical activity (PA) on the incidence of atrial fibrillation (AF) has been studied, contradictory results have been reported. Such discrepancies may reflect the different effects of various types of PA upon AF, as well as gender interactions. Therefore, we aimed to evaluate the associations of PA types (total, moderate/vigorous, and intentional), as well as walking pace, with AF risk in men and women.

**METHODS:**

Using the Multi-Ethnic Study of Atherosclerosis Typical Week Physical Activity Survey, 3 PA measures and walking pace were calculated among 6,487 men and women aged 45-84 years. The incidence of AF over approximately 11 years of follow-up was ascertained. The association of each PA measure and walking pace with AF incidence was estimated using multivariable Cox proportional hazard models. An extended Cox model with Heaviside functions (hv) of time was used to estimate the effects of time-varying covariates.

**RESULTS:**

During 11 years of follow-up (49,557 person-years), 242 new AF cases occurred. The incidence rate of AF was 48.83 per 10,000 person-years. The proportional hazard (PH) assumption for total PA among women was not met; hence, we used the hv to calculate the hazard ratio. Total PA in women in the hv2 analysis was negatively associated with AF in all 3 models, although for hv1 no significant association was observed. The PH assumption for walking pace among men was not met, and none of the hv showed a statistically significant association between walking pace and AF in men.

**CONCLUSIONS:**

These results suggest that PA is inversely associated with AF in women.

## INTRODUCTION

Atrial fibrillation (AF), the most common cardiac arrhythmia, is known to increase the risk of stroke (up to 5-fold), myocardial infarction, and heart failure [1-3]. The age-adjusted prevalence of AF in men and women in the 2010 Global Burden of Disease Study was estimated as 0.59 and 0.37%, respectively [4]. According to a projection study by Colilla et al. [5], in the US the prevalence of AF will more than double, from 5.2 million in 2010 to 12.1 million cases in 2030.

Favorable effects of physical activity (PA) on diseases such as obesity, hypertension, diabetes mellitus, coronary heart disease, and stroke have been identified [6-9]. However, it is unclear whether PA contributes to the incidence of AF. In fact, the impact of PA on the risk of AF remains controversial. Numerous studies have explored associations between PA and AF [10-15]. A systematic review and meta-analysis by Kwok et al. [16] indicated no significant association between intense PA and AF. In contrast, another systematic review and meta-analysis by Nielson et al. [17] reported a significant positive association between long-term vigorous PA and AF, while moderate PA was found to show a potential inverse correlation with AF incidence. These contradictory results may suggest that different types of PA have different effects on AF. Bertoni et al. [18], reported a significant association between both vigorous/moderate and intentional PA and the ankle-brachial index (ABI) in women. Moreover, walking pace, as a measure of the intensity of PA, was negatively associated with intima-media thickness (IMT) and ABI in both men and women. Therefore, it is plausible that the intensity of PA might affect AF.

In this cohort study, we aimed to evaluate the associations of PA types (total, moderate/vigorous, and intentional), as well as walking pace, with AF risk in men and women.

## MATERIALS AND METHODS

### Study population

The Multi-Ethnic Study of Atherosclerosis (MESA) is a population-based study in which 6,814 cardiovascular disease-free men and women aged 45-84 years were recruited. The participants were drawn from 6 communities in the US. Initially, its objective was to measure the incidence and prevalence of subclinical atherosclerosis based on indices such as the ankle-brachial index and the common and internal carotid IMT. The details have been presented elsewhere [19]. Written informed consent was provided by all participants.

In this follow-up study, 19 participants were excluded due to incomplete PA questionnaires. Moreover, 304 participants reported an illogical total amount of hours of activity per day (less than 0 or more than 24 hours per day), and were omitted. Four participants were excluded due to baseline angina and myocardial infarction. Ultimately, 6,487 participants were included in the analyses.

### Physical activity measurements

To measure PA, we used the MESA Typical Week Physical Activity Survey (TWPAS), a self-reported standardized questionnaire that has been validated in a cross-cultural activity participation study [20,21]. The TWPAS was designed to identify PA type and time during a typical week over the month preceding the survey. Various types of PA, including household, yard work, care, transportation, walking, dance, team sports, dual sports, individual activities, conditioning, leisure, and walking pace, were categorized into 3 levels of light, moderate, and heavy effort. The minutes of each activity were summed, converted to hours, and multiplied by metabolic equivalent of tasks (METs) [22].

We created 4 variables. The first was total PA (sum of all items, excluding walking pace). The second was the sum of 2 intensity levels (moderate and vigorous MET-hr/wk) for all items with these levels, except for walking pace. The third variable was created as intentional PA (sum of walking for exercise, sports/dancing, and conditioning MET-hr/wk). The fourth variable was walking pace in miles per hour (mph).

### Follow-up and ascertainment of atrial fibrillation

The follow-up period extended from July 15, 2000 through September 30, 2011 (approximation11 years), and was divided into 5 phases.

Initially, participants with self-reported AF were excluded from enrollment in MESA. Every 9-12 months, each MESA participant or a proxy was contacted to record all hospitalizations. An incident AF event was defined as a clinical modification code of 427.31 (AF) or 427.32 (atrial flutter) based on the International Classification of Diseases, 9th revision (during 2000-2011). Trained staff discarded AF cases associated with open surgery and adjudicated all AF events [23].

### Statistical analysis

Three PA measures—namely, total, intentional, and moderate/vigorous PA—were categorized into quartiles. Walking pace was created as a 3-level variable. A Cox proportional hazard (PH) model and an extended Cox model were used to examine the association of PA with AF incidence. Analysis of variance and the chi-square test were used for continuous and categorical variables, respectively. Age was used as the time scale to account for left-truncated survival time. In observational studies (such as MESA), subjects are already at risk for the outcome prior to their study entry. Hence, we used age as the time scale, and the outcome variable was age at event or censorship, which is a method that allows more effective control of age [24].

We generated 3 PA variables and walking pace and sought to investigate their independent associations with AF. For this purpose, 3 models were run. The first model was unadjusted (univariate model). Model 2 was adjusted for income, race, and pack-years of smoking. Model 3 was adjusted for the model 2 variables, as well as body mass index (BMI), alcohol, hypertension, diabetes, and lipid profile (total cholesterol and high-density lipoprotein [HDL]). In model 3, the variable of total PA was adjusted in order to assess the associations of walking pace, intentional PA, and moderate/vigorous PA with AF. A multiplicative interaction term was introduced to examine the possibility of interaction between PA measures and race. All analyses were stratified by gender. To test the PH assumption, the interaction with time and a graphical approach were utilized. In the men stratum, walking pace violated the PH assumption. In the women stratum, total PA violated the PH assumption. Because of this violation, we used the extended Cox (non-PH) model. For walking pace and total PA as non-PH variables in men and women, respectively, the Heaviside function (hv) was applied. For this reason, survival time was examined graphically for both walking pace in men and total PA in women. We noticed that the PH for walking pace survival time on day 800 (2.19 years) could be cut to make 2 hazard ratio (HR) values. However, the threshold for total PA was defined on day 400 (1.09 years). Using a single hv in the extended Cox model, 2 HR values would be applied, with each value considered constant during a fixed time interval. In essence, for walking pace in men, 2 HR values were calculated: the first for observations with a survival time of less than 800 days (2.19 years), and the second for observations with a survival time exceeding or equal to 800 days. Concerning total PA in women, 2 HR values were likewise esti - mated, but with a different threshold: the first for ob - servations with a survival time less than 400 days (1.09 years), and the second for observations with a survival time exceeding or equal to 400 days. All analyses were conducted using Stata version 12.0 (StataCorp., College Station, TX, USA).

### Ethical approval

For this study, we did not need ethical approval because the data were acquired under the National Heart, Lung, and Blood Institute (NHLBI)—Research Materials Distribution Agreement (RMDA) V02 1d20120806.

## RESULTS

This study consisted of 6,487 men and women (47.63% men). The mean age for the men and women was 62.33± 0.18 years and 62.40 ± 0.17 years, respectively. A total of 49,557 person-years were followed, during which 242 new AF cases occurred. The incidence rate of AF was calculated as 48.83 per 10,000 person-years.

The descriptive characteristics of the men and women subjects at baseline are presented in Table 1. All differences were statistically significant except for age, current alcohol use, race, and total PA (values for these variables were balanced across gender categories). As shown, moderate/vigorous and intentional PA were higher in men. Additionally, a higher percentage of men reported a fast walking pace ( > 3 mph), and the prevalence of diabetes was 3 percentage points higher (14.17%). Men were more likely to report both former and current smoking. BMI, total cholesterol, and HDL-cholesterol showed higher values in women.

Table 2 summarizes the crude frequency of AF by each PA measure and gender. Incident AF was less common in the upper quartiles of total PA in women. No other statistically significant associations were noted between incident AF and PA measures by gender stratification.

Table 3 summarizes the association between PA measures and incident AF by gender. Intentional and moderate/vigorous PA were unassociated with AF in either gender. In contrast to the lack of any significant association between total PA and AF for women in the hv1, we observed an inverse association between PA and AF for the hv2 in all three models. The HR for the category of 174-233 MET-hr/wk compared with the reference group of 5-122 MET-hr/ wk in the first model (univariate model) was calculated as 0.07 (95% confidence interval [CI], 0.01 to 0.61). The HRs for the second model (adjusted for income, race, and pack-year smoking) and the third model (adjusted for model 2 plus BMI, alcohol, hypertension, diabetes, total cholesterol, and HDL-cholesterol) were estimated as 0.06 (95% CI, 0.01 to 0.53) and 0.07 (95% CI, 0.01to 0.56), respectively. Moreover, the last category in the total PA among women (≥ 234 MET-hr/wk) in the hv2 analysis was also favorably associated with AF in all 3 models. In contrast, in men, only the last category (≥ 234 MET-hr/wk) in comparison to the reference group of 5-122 MET-hr/wk in the first model showed a protective effect against AF (HR, 0.47; 95% CI, 0.22 to 0.97).

The association between walking pace and AF is presented in Table 4. Two HR values for men before and after the point of 800 days were calculated. Walking pace was not associated with AF incidence in all 3 models for hv1 or for hv2. In addition, walking pace did not show a statistically significant association with AF incidence in women.

There was no evidence of multiplicative interaction between the 3 PA measures and walking pace and race/ethnicity in their associations with AF.

## DISCUSSION

In this prospective analysis, total PA was associated with reduced incident AF in women in the hv2 analysis (equal or exceeding 400 days’ survival time), even after controlling for demographic and lifestyle covariates. A study by Everett et al. [14], showed that PA in women may reduce the risk of AF; however, after controlling for BMI, this relationship disappeared. In our study, walking pace was not associated with AF incidence in women or men.

In a previous study, Bapat et al. [10], working with MESA data after a shorter follow-up (7.7 years), found no independent association between intentional and vigorous PA and the incidence of AF. However, in the vigorous PA subgroup, those with more intentional exercise showed a reduced risk of AF compared with those who did not engage in intentional exercise. Similarly, we did not observe any associations between intentional PA and AF. Our results regarding walking pace among men are in contrast with those of the study conducted by Mozaffarian et al. [15]. They found that walking pace was negatively associated with AF incidence in a study of older adults (≥ 65 years of age). Additionally, Williams & Franklin [11] reported that walking might reduce the risk of AF. They estimated that the risk of AF significantly decreased per MET-hr/d of walking. A systematic review by Zhu et al. [25] revealed that total PA had a direct effect on men and an indirect effect on women. Overall, they reported that PA is likely a risk factor for AF in men and a protective factor against AF in women, respectively. However, they indicated that neither total PA nor intensive PA, regardless of gender, was associated with AF incidence. Cardiac adaptation to PA may be different between men and women. Under comparable and equal conditions, men appear to exhibit deleterious structural remodeling [26]. In addition, compared with men, women produce fewer atrial electrophysiological changes in response to rapid atrial pacing [27]. Exercise is a very important mechanism that may lead to increased parasympathetic tone as a mediator of AF [13]. Moreover, certain mechanisms, regardless of gender, should be considered. Regular PA could disturb the balance between the sympathetic and parasympathetic nervous systems, leading to increased vagal tone, which could result in AF. Increasing the left atrial size is another mechanism that may be involved [28]. It should be noted that PA behavior is different between men and women. According to Keadle et al. [29], men engage in more and more vigorous PA and are more active than women.

Some limitations of the present study are evident. First, measurements of PA were self-reported, potentially leading to measurement bias (misclassification). Second, the power to find associations may not have been adequate, due in part to the presence of asymptomatic AF cases. Therefore, the desirable effects of PA may have been spoiled. Third, competing risk analysis was not applied to account for deaths during the follow-up period. Fourth, the TWPAS was designed to identify PAs and time spent in the typical week one month ago, and this “typical week” does not point to only a single time point. Instead, it refers to usual patterns of behavior. Thus, PA is not a momentary and temporary exposure. Additionally, PA is a time-varying variable and most likely changed within the follow-up period. As a result, repeated measurements are needed, and new analytical methods such as the g-formula and g-estimation should be applied.

Nonetheless, this study has some strengths. Using the hv for the predictors that violated the PH assumption improved the precision of our study. In fact, for PHs, it is not logical or permissible to report only a single HR value across the entire follow-up period; instead, it must be split. As another advantage, recruiting subjects belonging to a wide range of age groups (45-84 years) and different categories of race/ethnicity (Caucasian, Asian, African-American, and Hispanic) could enhance the generalizability of the study. In addition, by using adjusted models 2 and 3 (multivariable models), the influence of confounding was attenuated. In essence, confounding could exist because under some circumstances, some participants may increase their PA because of certain factors that increase the risk of AF.

In summary, these results revealed that gender may be an interactor in the association between total PA and AF incidence. Our findings suggest that PA is inversely associated with AF in women.

## Figures and Tables

**Table 1.** Characteristics of participants stratified by gender, MESA, United States, 2000-2011

Characteristics	Women (n = 3,397)	Men (n = 3,090)	p-value
Age (y)	62.40 士 0.17	62.33 士 0.18	0.78
Body mass index (kg/m^2^)	28.64 士 0.10	27.79 士 0.07	<0.001
Total cholesterol (mg/dL)	199.80 士 0.60	188.06 士 0.62	<0.001
HDLcholesterol (mg/dL)	56.32 士 0.26	45.06 士 0.21	<0.001
Alcohol, current use	1,650 (48.57)	1,937 (62.68)	0.19
Hypertension	1,590 (46.80)	1,334 (43.17)	0.003
Race/ethnicity			0.25
White	1,312 (38.62)	1,220 (39.48)	
Chinese	410 (12.07)	388 (12.56)	
Black	936 (27.55)	784 (25.37)	
Hispanic	739 (21.75)	698 (22.59)	
Annual income (US dollar)			<0.001
<50,000	2,187 (64.38)	1,581 (51.16)	
50,000-99,999	745 (21.93)	868 (28.09)	
≥100,000	334 (9.83)	527 (17.05)	
Diabetes	379 (11.15)	438 (14.17)	<0.001
Smoking			<0.001
Former	1,009 (29.70)	1,390 (44.98)	
Current	386 (11.36)	446 (14.43)	
Atrial fibrillation	95 (2.79)	147 (4.75)	<0.001
Walking pace (mph)			<0.001
Slow (<2)	1,034 (30.43)	764 (24.72)	
Medium (2-3)	1,710 (50.33)	1,546 (50.03)	
Fast (>3)	650 (19.13)	777 (25.14)	
Physical activity (MET-hr/wk)			
Total	187.58 士 1.46	186.00 士 1.74	0.48
Moderate/vigorous	75.58 士 1.12	96.89 士 1.57	<0.001
Intentional	20.44 士 0.49	26.70 士 0.61	<0.001

Values are presented as number (%) or mean ± standard deviation.

MESA, Multi-Ethnic Study of Atherosclerosis; MET, metabolic equivalent of task; mph, miles per hour (=1.6 km); HDL, high-density lipoprotein.

**Table 2.** AF by PA measures and walking pace, stratified by gender, MESA, United States, 2000-2011

	Women		Men	
	No. of participants	No. of AF	No. of participants	No. of AF
PA (MET-hr/wk)				
Total				
5-122	778	30	844	57
123-173	889	33	746	33
174-233	878	21	731	31
≥ 234	852	11^*^	769	26
Moderate/vigorous				
0-34	936	12	693	11
35-69	902	11	723	16
70-139	878	5	749	16
≥ 140	681	3	925	16
Intentional				
None	851	10	671	12
1-14	1,122	11	825	18
15-29	651	5	628	10
≥ 30	773	5	966	19
Walking pace (mph)				
Slow (<2)	1,034	37	764	45
Medium (2-3)	1,710	46	1,546	68
Fast (>3)	650	12	777	34

MESA, Multi-Ethnic Study of Atherosclerosis; AF, atrial fibrillation; PA, physical activity; MET, metabolic equivalent of task; mph, miles per hour (= 1.6 km).

* p<0.05 for comparisons across categories.

**Table 3.** Association between physical activity (MET-hr/wk) measures and atrial fibrillation, stratified by gender, MESA, United States, 2000-2011

PA	Women						Men		
Intentional^1^	1-140		15-29		≥30		1-14	15-29	≥30
Model 1^2^	0.80 (0.34, 1.88)		0.62 (0.21, 1.81)		0.51 (0.17, 1.51)		1.15(0.55,2.40)	0.83 (0.36,1.93)	1.05 (0.51,2.16)
Model 2^3^	0.74 (0.31, 1.77)		0.57 (0.19, 1.70)		0.47 (0.16, 1.41)		1.18(0.55,2.53)	0.91 (0.38,2.16)	1.06 (0.49,2.29)
Model 3^4^	0.69 (0.29, 1.65)		0.54 (0.18, 1.63)		0.53 (0.17, 1.66)		1.28 (0.59,2.77)	1.02 (0.42,2.44)	1.27(0.57,2.83)
Moderate/vigorous^5^	35-69		70-139		≥140		35-69	70-139	>140
Model 1^2^	0.89 (0.39. 2.02)		0.39 (0.14, 1.13)		0.31 (0.08, 1.12)		1.29 (0.60,2.78)	1.24 (0.57,2.67)	0.97 (0.45,2.11)
Model 2^3^	0.85 (0.37, 1.94)		0.36 (0.12, 1.04)		0.31 (0.08, 1.11)		1.38 (0.62,3.09)	1.36 (0.61,3.06)	1.06 (0.47,2.38)
Model 3^4^	0.83 (0.34, 2.01)		0.43 (0.12, 1.50)		0.46 (0.07, 2.83)		1.65 (0.72,3.75)	1.87 (0.76,4.60)	2.24 (0.71,7.08)
Total^6^	hv1^7^ (< 400 d survival time)			hv2^8^ (≥400 d survival time)			123-173	1 74-233	>234
	123-173	174-233	>234	123-173	174-233	>234			
Model 1^2^	0.81 (0.16,4.06)	1.08 (0.24,4.83)	0.81 (0.16,4.03)	0.48 (0.17,1.33)	0.07 (0.01,0.61)	0.08 (0.01,0.63)	0.67 (0.35,1.31)	0.51 (0.24,1.05)	0.47 (0.22,0.97)
Model 2^3^	0.70 (0.14,3.51)	0.92 (0.20,4.16)	0.73 (0.14,3.63)	0.42 (0.15,1.17)	0.06 (0.01,0.53)	0.07 (0.01,0.57)	0.69 (034,1.37)	0.59 (0.24,1.11)	0.49 (0.23,1.05)
Model 3^4^	0.58 (0.11,2.94)	0.80 (0.17,3.64)	0.62 (0.12,3.13)	0.40 (0.14,1.14)	0.07 (0.01,0.56)	0.07 (0.01,0.59)	0.77 (038,1.54)	0.57(0.26,1.25)	0.59 (0.27,1.28)

MESA, Multi-Ethnic Study of Atherosclerosis; MET, metabolic equivalent of task; hv, Heaviside function.

1 Reference category: no MET-hr/wk.

2 Model 1 was unadjusted (univariate model).

3 Model 2 was adjusted for race, income, and pack-years of smoking.

4 Model 3 was adjusted for model 2 variables plus body mass index, alcohol, hypertension, lipids (total cholesterol, high-density lipoprotein cholesterol), and diabetes.

5 Reference category: 0-34 MET-hr/wk.

6 Reference category: 5-122 MET-hr/wk.

7 hv1: for the observations with a survival time less than 1,200 days (3.28 years).

8 hv2: for the observations with a survival time exceeding or equal to 1,200 days (3.28 years).

**Table 4.** Association between walking pace (mph)^1^ and atrial fibrillation, stratified by gender, MESA, United States, 2000-2011

	Women		Men			
	Medium (2-3)	Fast (>3)	hv1^5^ (< 800 d survival time)		hv2^6^ (≥ 800 d survival time)				
			Medium (2-3)	Fast (>3)	Medium (2-3)	Fast (>3)		
Model 1^2^	0.61 (0.27, 1.33)	0.72 (0.27, 1.92)	1.31 (0.61, 2.80)	0.57 (0.20, 1.62)	1.17 (0.31, 4.44)	1.92 (0.49, 7.46)
Model 2^3^	0.59 (0.26, 1.32)	0.68 (0.24, 1.86)	1.42 (0.64, 3.16)	0.61 (0.21, 1.78)	1.50 (0.31, 7.25)	2.35 (0.47, 11.73)
Model 3^4^	0.59 (0.26, 1.36)	0.69 (0.23, 2.00)	1.67 (0.75, 3.75)	0.76 (0.26, 2.27)	1.69 (0.34, 8.22)	2.85 (0.56, 14.13)

MESA, Multi-Ethnic Study of Atherosclerosis; mph, miles per hour (= 1.6 km); hv, Heaviside function.

1 Reference category: slow (<2 mph).

2 Model 1 was unadjusted (univariate model).

3 Model 2 was adjusted for race, income, and pack-years of smoking.

4 Model 3 was adjusted for model 2 variables plus body mass index, alcohol, hypertension, lipids (total cholesterol, high-density lipoprotein cholesterol), and diabetes.

5 hv1: for the observations with a survival time less than 800 days (2.19 years).

6 hv2: for the observations with a survival time exceeding or equal to 800 days (2.19 years).
